# The effect of dexmedetomidine-ketamine combination versus dexmedetomidine on behavior of uncooperative pediatric dental patients: a randomized controlled clinical trial

**DOI:** 10.1590/1678-7757-2024-0057

**Published:** 2024-09-20

**Authors:** Sara Hassan El-ROUBY, Yasmi O CRYSTAL, Ahmed M ELSHAFIE, Nadia A WAHBA, Magda M El-TEKEYA

**Affiliations:** 1 Alexandria University Faculty of Dentistry, Pediatric Dentistry Dental Public Health Department Egypt Alexandria University, Faculty of Dentistry, Pediatric Dentistry and Dental Public Health Department, Egypt.; 2 NYU College of Dentistry Department of Pediatric Dentistry USA NYU College of Dentistry, Department of Pediatric Dentistry, USA.; 3 Alexandria University Faculty of Medicine Egypt Alexandria University, Faculty of Medicine, Egypt.

**Keywords:** Dexmedetomidine, Ketamine, Moderate Sedation, Child behavior, Oral administration

## Abstract

**Objective:**

Uncooperative behavior in pediatric dentistry is one of the most common manifestations of dental anxiety. Managing anxious patients can be attained by moderate sedation. This study aimed to compare the effect of sedation by dexmedetomidine-ketamine combination (DEX-KET) versus dexmedetomidine (DEX) on behavior of uncooperative pediatric dental patients.

**Methodology:**

In total, 56 uncooperative healthy children (3–5 years old) requiring dental treatment were divided randomly into two groups: Group I (study group), which received buccal dexmedetomidine (2 μg/kg) and ketamine (2 mg/kg), and Group II (control group), which received only buccal dexmedetomidine (4 μg/kg). Drugs effects were assessed in terms of hemodynamic parameters, patient’s drug acceptance, child behavior, postoperative effect of sedation, amnesic effect, incidence of adverse events, as well as procedural induced stress measured by salivary secretory immunoglobulin A (s-IgA).

**Results:**

Hemodynamic results did not reveal a statistically significant difference between the two study groups (P>0.05). There was a significant difference in patient’s acceptance to sedative drug between both groups, favoring DEX (p=0.005). Children who received DEX-KET showed significantly better behavior than those who received DEX for local anesthesia (p=0.017) and during operative procedure (p=0.037). Adverse events, post-operative and amnesic effects of drugs were comparable in both groups (p>0.05). Moreover, the mean difference in the salivary s-IgA levels between initial and final value was not statistically significant between both groups (p=0.556).

**Conclusion:**

Both DEX-KET combination and DEX alone are effective in providing hemodynamic stability. DEX-KET combination significantly improved the behavior of sedated children compared to DEX alone but the drug acceptance was decreased in the DEX-KET group. Both regimens did not have a negative effect on postoperative behavior of children and had comparable amnesic effect with no significant adverse events. Salivary s-IgA is not considered a potential stress biomarker in sedated children.

## Introduction

Uncooperative behavior is one of the most prevalent manifestations of dental anxiety in children and may lead to delaying or deferring treatment or to a decrease in the quality of dental care. Managing behavior of uncooperative children can be challenging and, in certain situations, unattainable by using basic behavior management techniques.^1^ The use of moderate sedation induces a more positive behavior and allows for the necessary provision of care in a compassionate manner. Pharmacological agents used must reduce risks and prevent complete loss of consciousness. Safe practice requires matching drug selection to type of the procedure and minimizing number of drugs selected.^2^ In pediatric dentistry, several sedative drugs have been used via various routes for moderate sedation. Each administration path shows its own benefits and drawbacks.

Dexmedetomidine is an alpha-2 agonist and considered a promising sedative agent for pediatric patients. It was initially approved by the U.S. Food and Drug administration (FDA) in 1999 for premedication and sedation of patients in intensive care units. It shows sedative and analgesic properties that control pain, stress, and anxiety. In pediatric patients, it results in stable respiratory rate and predictable cardiovascular reactions.^3^ However, dexmedetomidine presents some disadvantages such as slow onset, induced bradycardia, and hypotension.^4^

Ketamine is a phencyclidine derivative, used in children due to its analgesic, amnesic, and hypnotic effect. It is highly effective and shows a great safety profile, preserving airway reflexes, and spontaneous ventilation.^5^ Its disadvantages include side effects of irritability, induced tachycardia, and hypertension. It is advisable to administer ketamine in conjunction with other agents to reduce its adverse effects. The drawbacks of dexmedetomidine and ketamine can both be counterbalanced when used in combination.^6^

Dexmedetomidine and ketamine combination can be administered intramuscularly, intravenously, or transmucosally. The transmucosal drug delivery system includes rectal, intranasal, buccal, or sublingual routes. Buccal route of administration, using an atomizer to deliver the drug on the buccal pouch, offers a rapid onset and bypasses the first-pass metabolism compared to oral sedation. It is considered as a painless, non-invasive procedure, being favored by children.^7^

Children may become apprehensive during dental treatment due to various stimuli, which frequently triggers the onset of psychological or physical stress.^8^ Although studies have attempted to assess stress during dental treatment using various questionnaires or physiological indices, such as blood pressure and heart rate,^8^ it is difficult to objectively measure the latent stress of dental treatment in children. Saliva has recently drawn attention as a sample for stress-related substances measurement since its collection is less invasive, safer, and easier than blood sampling. Saliva contains catecholamines, cortisol, salivary amylase, chromogranin A (CgA), and secretory immunoglobulin A (s-IgA), among other chemicals related to stress.^9^ Acute stressors can increase salivary s-IgA within 5–6 minutes after their initiation, whereas cortisol, the most widely used stress biomarker, peaks around 20–30 mins after exposure. In addition, s-IgA shows an added advantage over cortisol since it exhibits a quick decrease during recovery at approximately 30 minutes.^10^ To the best of our knowledge, no study has investigated the salivary s-IgA as stress biomarker in sedated children undergoing dental treatment.

Recent literature supports that dexmedetomidine and ketamine work synergistically, which could lead to a reduction in the dosage of both sedatives.^11,12^ However, there is insufficient evidence on the effect of the combination of dexmedetomidine and ketamine on the behavior of children undergoing dental treatment.^13^ Therefore, this study aims to compare the effect of sedation by dexmedetomidine-ketamine combination (DEX-KET) versus dexmedetomidine (DEX) on behavior of uncooperative pediatric dental patients using the buccal route of administration.

## Methodology

### Study design

This study was a two-arm randomized clinical trial with a parallel design. It was designed and reported following the Consolidated Standards of Reporting Trials (CONSORT) guidelines.^14^ The Dental Research Ethics Committee of the Faculty of Dentistry granted the study ethical approval (IRB NO: 00010556 – IORG 0008839), and registered it in the Pan African Clinical Trials Registry database (pactr.samrc.ac.za PACTR202105602764595) before the trial or patient enrollment. Patient recruitment and data collection were conducted from August 2022 to March 2023. Guardians of all children were asked to sign an informed consent form after being given a detailed explanation about potential risks and benefits involved in the study.

### Sample size estimation

Sample size was estimated assuming 5% alpha error and 80% study power. The percentage of ease of treatment completion was 93.8% for dexmedetomidine-ketamine^15^ and 61.54% for dexmedetomidine.^16^ Based on the difference between independent proportions, a sample size of 28 children per group was obtained, with a total sample of 56 children. The sample size was estimated using G*Power software (version 3.1.97).

### Study sample

Study subjects included 56 healthy children aged 3–5 years who attended the outpatient clinic at the Pediatric Dentistry and Dental Public Health Department, Faculty of Dentistry, Alexandria University, for whom basic behavior management techniques had been unsuccessful to deliver the necessary dental care. Patients included were healthy children free from any systemic disease, categorized in the American Society of Anesthesiologists (ASA) as Class I or II.^17^ Participating children exhibited definitely negative and negative behavior (Frankl score 1 or 2)^18^ and needed dental treatment under local anesthesia that could be completed in less than 30 minutes. Exclusion criteria included known hypersensitivity or allergy to any of the test drugs, medically or cognitively compromised patients and children who needed extensive dental treatment requiring general anesthesia. Children were recruited after presenting the study protocol to their parents and obtaining informed consent.

### Grouping, randomization technique, and allocation concealment

Enrolled children were randomly assigned to one of the two groups using a computer-generated list of random numbers:

Group I (n = 28) patients received buccal aerosolized dexmedetomidine and ketamine combination (DEX- KET). In total, 2μg/kg DEX and 2 mg/kg KET.^19^

Group II (n = 28) patients received buccal aerosolized dexmedetomidine alone (DEX). In total, 4μg/kg DEX.^20^

Allocation was performed by using permuted block technique, with equal allocation ratio using random allocation software. Each allocation was represented by a code (the serial of the participant in the study and the group name), sealed in serially numbered opaque envelopes and delivered to the pediatric anesthesiologist (PhD), responsible for all anesthetic-related procedures in participants.^21^

### Blinding

The researcher (pediatric dentist performing all operative procedures and assessment of all the study outcomes), participants, and statistician were blinded to the drug regimen administered (triple blind). Only the main supervisor and the anesthesiologist were familiar with the allocation group. After data collection, the allocation group was revealed by breaking the randomization code.

### Intra-examiner reliability

Weighted Kappa Coefficient was used to estimate the intra-examiner reliability, which was included watching videotapes of a group of 15 patients twice with six days interval between the first and second views.^22^ K was found to be 0.88 for children behavior using Ohio State University Behavior Rating Scale (OSUBRS). These patients were not involved in the clinical trial.^23^

### Patient preparation

**Figure 1:**
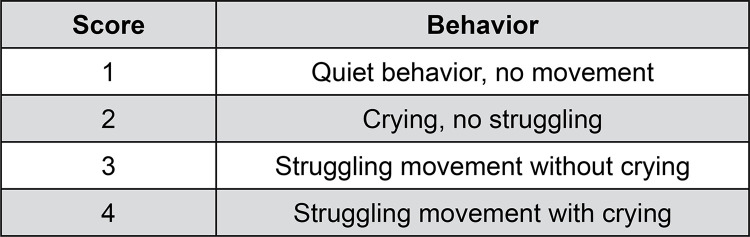
Ohio State University behavior rating scale.^21^

A brief medical history was obtained from the guardian on the day before the dental procedure. Eligible patients were examined by the anesthesiologist to assess if they were fit for sedation and guardians were informed about the pre-sedation fasting instructions of 2, 4, and 6h of fasting for clear liquids, milk, and light meals, respectively.^24^ Child’s behavior was assessed at baseline following the OSUBRS (Figure 1).^23^

On the day of the appointment, vital parameters were monitored for all participants before, during treatment, and until discharge, including systolic and diastolic blood pressure (SBP and DBP, respectively) using a digital sphygmomanometer (Joytech Healthcare DBP-1231 Arm-type Fully Automatic Digital Blood Pressure Monitor ), as well as heart rate and oxygen saturation using a pediatric pulse oximeter (CONTEC CMS50D Fingertip Pulse Oximeter).

### Assessment of procedural induced stress (Salivary s-IgA)

The first saliva sample was collected from each child while in the waiting room before drug administration using a sterile cotton roll placed sublingually for 1 minute until it soaked to collect 0.5 ml of saliva. This was inserted into a syringe, then saliva was ejected into an Eppendorf tube labeled with the code number of the patient and stored at a temperature of −80°C until analyzed. The second salivary sample was collected post operatively using the same methods. For analysis, samples were thawed and centrifuged for 10 minutes at 2,000–3,000 rpm at 2–8°C to remove particulates. Salivary s-IgA was measured using an enzyme-linked immunosorbent assay ELISA kit (Sinogeneclon Co., Ltd Human IgA Elisa Kit: Catalog No-SG-1022; Size- 96 Microwells; Lot No- 20221110; China) following the manufacturer instructions.^25^

### Sedation procedure

Child’s body weight was recorded at the start of the appointment to estimate the proper dose of the sedative drugs. The drugs were prepared from parenteral forms of ketamine (Ketamine 50 mg / ml, Rotexmedica, Trittau, Germany) or dexmedetomidine (Precedex 200 mcg/2ml, Hospira. Inc., Lake Forest, IL USA) with saline 0.9% added to obtain the final volume. In Group I, each drug was loaded in a different syringe. Meanwhile, in Group II, the drug dosage was divided into two identical syringes. This procedure was performed to keep the intervention group blinded.

**Figure 2:**
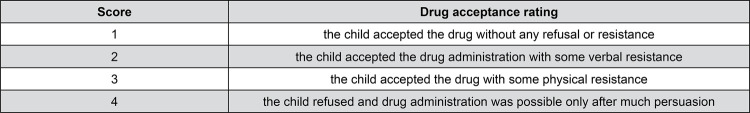
Child’s acceptance to the drug using the 4-point rating scale.^25^

The drugs were administered by the anesthesiologist using the LMA MAD Nasal mucosal atomization device (Teleflex, Inc., Research Triangle Park, NC) connected to a 2 ml / 5 ml syringe, which transforms the solution into a fine mist, enabling uniform drug delivery.^15^ The MAD was explained to the children with the tell-show-do technique, for them to understand and follow the drug administration instructions without swallowing it. The sedative drugs were administered via the buccal mucosa or in the buccal pouch, evenly divided and sprayed into both cheeks.^26^ The child’s acceptance to the drug administered was assessed by the anesthesiologist using a 4-point rating scale (Figure 2).^27^ To assess anterograde amnesic effect of the sedative drugs, the child was shown a picture (Pic1: Apple) and was asked to verbally identify it immediately after drug administration (encoding Phase I).^28^

### Operative procedure

The operative procedure started once the child reached the drowsy or asleep stage following the Wilton, et al. sedation scale, considered a state of optimum sedation.^29^ For assessing the anterograde amnesic effect, a second picture (Pic2: train) was shown to the child, who was asked to verbally identify it (Phase1; encoding Phase II) immediately before the local anesthesia administration.^28^ Topical anesthesia (benzocaine 30 mL 20%) (Opahl-S, DHARMA RESEARCH INC., USA.) was applied for 60 seconds after drying the tissues. The dose of local anesthetic (Articaine HCL 4% and epinephrine 1:100,000) (ARTINIBSA 4%, Inibsa Dental S.L.U., Spain) was estimated according to patients’ weight.^30^ Dental treatment (Simple restorations, Pulpotomy, Stainless Steel Crown, Extraction) was standardized to be accomplished in 15–30 minutes for all patients enrolled in the study. Before discharge, the final stage of the assessment of anterograde amnesia was performed by asking the child to identify the two pictures previously shown in the encoding phases among four pictures; two target pictures (Pic1: Apple, Pic2: Train) and two distractor pictures (Pic3: Carrot, Pic4: Motorbike). This was regarded as Phase II: recall phase.^28^ Children were discharged after meeting the discharge criteria following the American Academy of Pediatric Dentistry (AAPD) guidelines.^24^ The sedation duration was assessed from the onset of sedation till complete recovery of the patient. Common procedural side effects, including agitation, bradycardia, hypotension, vomiting, and others, observed throughout the procedure were documented.

### Post-operative evaluation

The whole procedure was videotaped, and child behavior was assessed by the operator for both groups using the OSUBRS by watching the recorded videos and attributing behavior scores at local anesthesia administration and during the operative procedure. After 24 hours, parents were contacted by phone to answer the modified Vernon, Schulman, and Foley^31^ (1966) questionnaire regarding the postoperative response of their children.

### Statistical analysis

The Mann Whitney U test was applied for comparisons between groups regarding patient’s acceptance of drugs, children behavior during sedation, and postoperative child behavior questionnaire. Pearson’s chi-square and Fisher’s exact test were used to assess differences between groups in amnesic effects of sedative agents. Independent t test was used to compare salivary IgA between groups whereas paired t test was used to assess differences in salivary IgA before and after intervention. All tests were two tailed and the significance level was set at p≤0.05. Data were analyzed using the IBM SPSS, version 23, Armonk, NY, USA.

## Results

**Figure 3:**
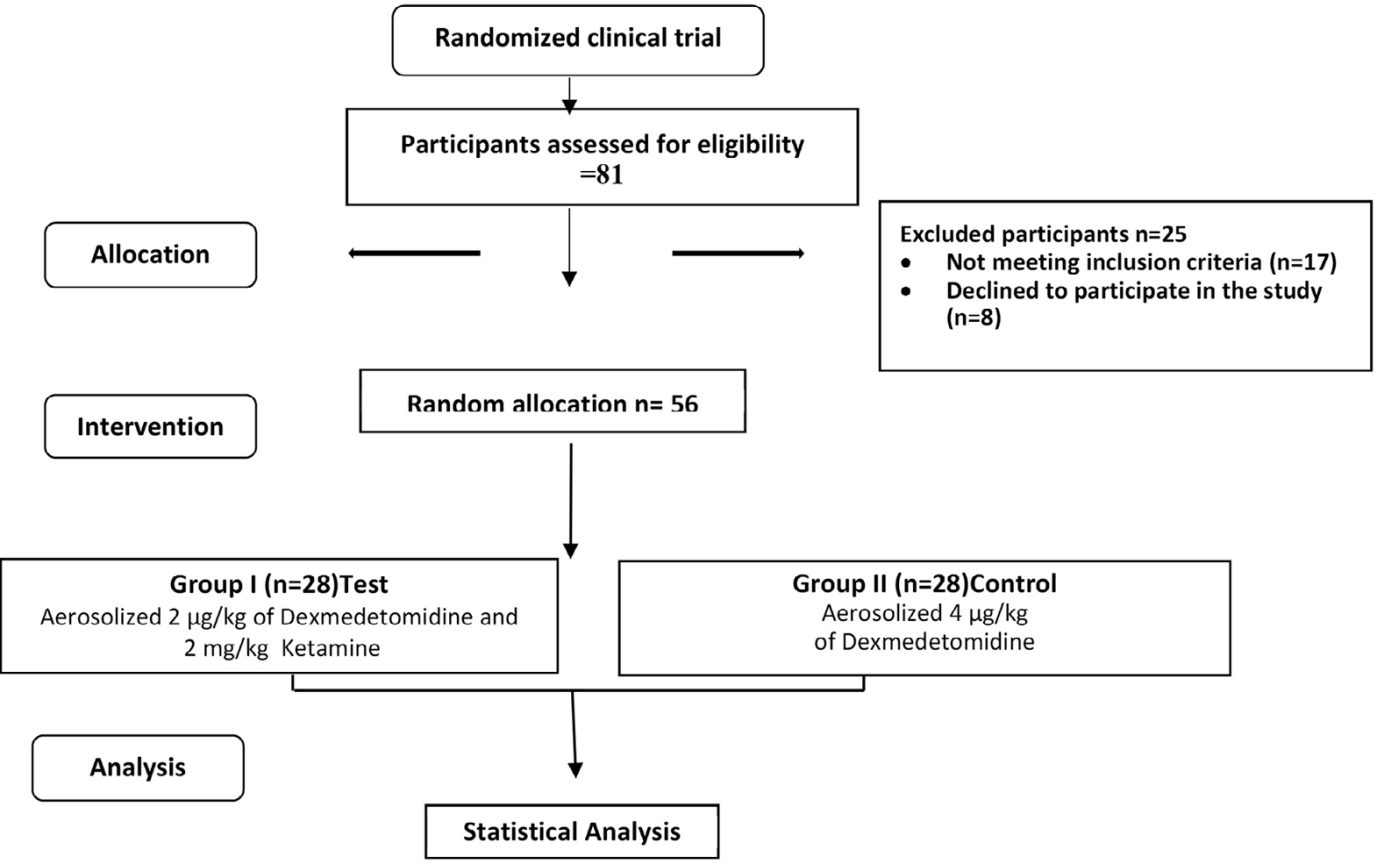
CONSORT diagram showing the study protocol

From 81 children inspected for eligibility, 56 were recruited and randomly allocated to either the DEX-KET group or DEX group (Figure 3). The mean age of selected children was 4.28 ± 0.63, with 27 males (48%) and 29 females (52%). Hemodynamic results showed no statistically significant differences between the two study groups regarding: systolic blood pressure, diastolic blood pressure, heart rate, and oxygen saturation (p>0.05) (Table 1). Sedation duration was significantly shorter in the DEX-KET group (40.23±8.08) minutes when compared to DEX group (55.15±13.32) minutes (p<0.0001). Regarding the drug acceptance, results showed a significantly higher acceptance in the DEX group compared to the DEX-KET combination (p*=*0.005) (Table 2 and Table 3). We found no significant differences in patient’s behavior at baseline between both groups assessed by OSUBRS (p=0.065). However, we found a statistically significant improvement in patient’s behavior scores in favor of the DEX-KET at local anesthesia administration (p=0.017) and during the operative procedure (p=0.037). The improvement in the behavior scores from baseline was significant in both groups (p<0.0001) (Table 4). Drug-related adverse effects were comparable in both groups with no statistically significant differences (p>0.05). Regarding the amnesic effect of sedative drugs, we found no statistically significant differences between the study groups (Table 5). Moreover, we noted no significant differences between salivary s-IgA levels before and after treatment in both DEX-KET and DEX groups (p=0.535, p=0.739, respectively) (Table 6). Furthermore, the mean difference in salivary s-IgA levels between initial and final value was not statistically significant between groups (p=0.556) (Table 6). We found no statistically significant differences for post-operative effects of sedation considering the scores obtained in the Vernon, Schulman, and Foley (1966) modified questionnaire,^31^ as parents reported that children’s behavior was same as before (p>0.05) (Table 7).

**Table 1:** Comparison of hemodynamic parameters between the study groups

		Dexmedetomidine + Ketamine	Dexmedetomidine	p value¥
		**(n=28)**	**(n=28)**	
		**Mean ± SD**		
SPo2	Baseline	98.00±0.98	98.35±0.85	0.179
	During	98.08±0.80	98.04±0.82	0.865
	After	98.73±0.53	98.58±0.58	0.323
Heart Rate (HR)	Baseline	104.92±16.30	107.62±15.10	0.539
	During	104.38±12.50	104.85±13.55	0.899
	After	103.31±13.66	100.27±10.47	0.372
Systolic blood pressure (SBP)	Baseline	100.85±16.11	95.73±14.90	0.240
	During	95.35±16.18	98.46±18.67	0.523
	After	93.35±17.29	92.38±19.62	0.852
Diastolic blood pressure (DBP)	Baseline	69.96±14.35	64.38±14.49	0.169
	During	64.96±12.93	65.85±16.50	0.830
	After	60.50±13.59	60.23±11.58	0.939

¥According to the independent t test.

**Table 2:** Comparison of patient’s drug acceptance scores between the study groups

scores	Dexmedetomidine + Ketamine (n=28)	Dexmedetomidine (n=28)	p value¥
Median (IQR)	2.00 (2.00)	1.00 (0.75)	0.005*
Min - Max	1.0 – 4.00	1.00 – 3.00	

*Statistically significant difference at p value<0.05, ¥According to the Mann Whitney U Test.

**Table 3:** Distribution of drug acceptance scores in both groups

Drug acceptance scores	Dexmedetomidine + Ketamine (n=28)	Dexmedetomidine (n=28)
	**n (%)**	
Score 1 (good)	11 (39.3%)	21 (75%)
Score 2 (Fair)	8 (28.6%)	4 (14.3%)
Score 3 (poor)	5 (17.9%)	3 (10.7%)
Score 4 (refused)	4 (14.3%)	0 (0%)

**Table 4:** Comparison of patients’ behavior scores using the OUSBRS at baseline, during local anesthesia, and during treatment.

Time points	Scores	Dexmedetomidine + Ketamine (n=28)	Dexmedetomidine (n=28)	p value¥
Baseline	Median (IQR)	3.00 (1.00)	4.00 (1.00)	0.065
	Min - Max	2.00 – 4.00	2.00 – 4.00	
At local Anesthesia	Median (IQR)	2.00 (3.00)	4.00 (2.00)	0.017*
	Min - Max	1.00 – 4.00	1.00 – 4.00	
During treatment	Median (IQR)	2.00 (2.00)	3.00 (2.75)	0.037*
	Min - Max	1.00 – 4.00	1.00 – 4.00	
p value§		<0.0001*	<0.0001*	
Pairwise comparisons		P1=0.002*, P2<0.0001*, P3=1.00	P1=0.158, P2=0.012*, P3=1.00	

*Statistically significant difference at p value<0.05, ¥According to the Mann Whitney U Test, §According to the Friedman Test.

**Table 5:** Comparison of amnesic effect of sedative agents between the study groups

	Scores	Dexmedetomidine + Ketamine (n=28)	Dexmedetomidine (n=28)	p value¥
		**n (%)**		
Picture I	Identified	21 (75%)	25 (89.3%)	0.163
	Not identified	7 (25%)	3 (10.7%)	
Picture II	Identified	22 (78.6%)	25 (89.3%)	0.469
	Not identified	6 (21.4%)	3 (10.7%)	

¥According to the Fisher’s Exact Test.

**Table 6:** Comparison of salivary sIgA levels between the study groups

Time points	Dexmedetomidine + Ketamine (n=28)	Dexmedetomidine (n=28)	p value
Before: Mean ± SD (μg/ml)	5.63±1.93	6.33±1.73	0.178ⱡ
After: Mean ± SD (μg/ml)	5.37±1.18	6.21±1.13	0.011*ⱡ
p value§	0.535	0.739	
Difference: Mean ± SD (μg/ml)	0.27 (2.18)	0.12 (1.75)	0.556¥

*Statistically significant difference at p value<0.05, According to the independent t test, ¥According to the Mann Whitney U Test. §According to the paired t test.

**Table 7:** Comparison of child behavior questionnaire responses between the study groups

		Dexmedetomidine + Ketamine (n=28)	Dexmedetomidine (n=28)	p value¥
Does your child make a fuss about going to bed at night?	Less than before	0 (0%)	0 (0%)	1.00
	Same as before	28 (100%)	28 (100%)	
	More than before	0 (0%)	0 (0%)	
Does your child make a fuss about eating?	Less than before	2 (7.1%)	3 (10.7%)	0.638
	Same as before	26 (92.9%)	25 (89.3%)	
	More than before	0 (0%)	0 (0%)	
Does your child wet the bed at night?	Less than before	0 (0%)	0 (0%)	1.00
	Same as before	28 (100%)	28 (100%)	
	More than before	0 (0%)	0 (0%)	
Does your child bite their fingernails?	Less than before	0 (0%)	0 (0%)	1.00
	Same as before	28 (100%)	28 (100%)	
	More than before	0 (0%)	0 (0%)	
Does your child get upset when you leave them alone for a few minutes?	Less than before	0 (0%)	0 (0%)	1.00
	Same as before	28 (100%)	28 (100%)	
	More than before	0 (0%)	0 (0%)	
Does your child need a lot of help with tasks?	Less than before	0 (0%)	0 (0%)	1.00
	Same as before	28 (100%)	28 (100%)	
	More than before	0 (0%)	0 (0%)	
Does your child seem to avoid or fear new things?	Less than before	0 (0%)	0 (0%)	1.00
	Same as before	28 (100%)	28 (100%)	
	More than before	0 (0%)	0 (0%)	
Does your child have temper tantrums?	Less than before	0 (0%)	0 (0%)	1.00
	Same as before	28 (100%)	28 (100%)	
	More than before	0 (0%)	0 (0%)	
Is it difficult to get your child to talk to you?	Less than before	0 (0%)	0 (0%)	1.00
	Same as before	28 (100%)	28 (100%)	
	More than before	0 (0%)	0 (0%)	
Does your child follow you around the house?	Less than before	0 (0%)	0 (0%)	1.00
	Same as before	28 (100%)	28 (100%)	
	More than before	0 (0%)	0 (0%)	
Does your child spend time trying to get or hold your attention?	Less than before	0 (0%)	0 (0%)	1.00
	Same as before	28 (100%)	28 (100%)	
	More than before	0 (0%)	0 (0%)	
Is your child afraid of the dark?	Less than before	0 (0%)	0 (0%)	1.00
	Same as before	28 (100%)	28 (100%)	
	More than before	0 (0%)	0 (0%)	
Does your child have bad dreams or wake up crying at night?	Less than before	0 (0%)	0 (0%)	1.00
	Same as before	28 (100%)	28 (100%)	
	More than before	0 (0%)	0 (0%)	
Does your child have trouble falling asleep at night?	Less than before	0 (0%)	0 (0%)	1.00
	Same as before	28 (100%)	28 (100%)	
	More than before	0 (0%)	0 (0%)	
Does your child seem shy or afraid around strangers?	Less than before	0 (0%)	0 (0%)	1.00
	Same as before	26 (100%)	26 (100%)	
	More than before	0 (0%)	0 (0%)	
Does your child have a poor appetite?	Less than before	0 (0%)	0 (0%)	0.641
	Same as before	26 (100%)	26 (100%)	
	More than before	0 (0%)	0 (0%)	
Does your child tend to disobey you?	Less than before	0 (0%)	0 (0%)	1.00
	Same as before	26 (100%)	26 (100%)	
	More than before	0 (0%)	0 (0%)	
Does your child break toys or other objects?	Less than before	0 (0%)	0 (0%)	1.00
	Same as before	26 (100%)	26 (100%)	
	More than before	0 (0%)	0 (0%)	

¥Comparison was done using Mann Whitney U Test.

## Discussion

Managing uncooperative and anxious children during pediatric dental treatment may be unachievable by the traditional behavioral management techniques. For this reason, moderate sedation has been increasingly used to improve child cooperation and enable the delivery of excellent quality of dental care. Various drugs have been used to achieve this goal and, in a trial, aiming to improve the results, new combinations with various routes of administrations have been proposed.^7^ Dexmedetomidine and ketamine exhibit complementary pharmacological effects. When used together, dexmedetomidine may attenuate the tachycardia, hypertension, and salivation, emergent phenomena associated with ketamine. This combination shows low incidence of side effects and rapid recovery with no cardiorespiratory depression.^11^ However, to date, to the best of our knowledge, a comparative study of buccal administration of DEX-KET combination and DEX for sedation of pediatric dental children has not been published. Thus, a well-designed randomized controlled clinical trial is needed to evaluate and compare the effect of DEX-KET combination versus DEX on behavior and anxiety of uncooperative children requiring dental treatment. This study showed that both DEX-KET combination and DEX alone were effective in promoting hemodynamic stability. Moreover, DEX-KET combination provided rapid recovery compared to DEX alone. Previous studies have reported that dexmedetomidine-ketamine combination showed complementary pharmacological effects, as these medications hold opposing hemodynamic effects, and the addition of KET to DEX offered rapid recovery when compared to dexmedetomidine alone.^12,32^ In this study, patients in DEX group showed significantly better drug acceptance than the DEX-KET group. This could be attributed to the fact that dexmedetomidine is tasteless and odorless, which helped the children accept the drug and keep it for 30 seconds in the mouth.^33^ On the other hand, the ketamine formulation has a bitter and astringent taste.^34^ The results of this study show that children in the dexmedetomidine-ketamine group showed a quieter attitude throughout local anesthesia injection and during the operative procedure, when compared to those who received dexmedetomidine alone, as assessed by the OSUBRS. It therefore appears that combining the anxiolytic effects of dexmedetomidine and analgesic effects of ketamine could improve pediatric behavior compared to using DEX alone. The superiority of combining dexmedetomidine and ketamine has been demonstrated in previous studies, which reported that this combination can improve child behavior during parental separation and venous cannulation.^12,35^ Our results also corroborate a previous study by Agarwal, et al.^15^ (2023), who confirmed that the frequency of calm and cooperative behavior was higher in the DEX-KET group when compared to different drugs combinations. On the contrary, Sado-Filho, et al.^36^ (2021) found no significant differences in the percentage of calm behavior between dexmedetomidine alone and in combination with ketamine when assessing procedural sedation for pediatric dental settings. The difference in their results could be explained by their use of different intranasal drug dosages. Another study by Haider, et al.^37^ (2022) reported that DEX alone was equally efficacious to DEX-KET combination for sedation of uncooperative pediatric dental patients. This could be attributed to the administration at different dosages via intravenous route followed by a maintenance dose to keep children sedated throughout the whole procedure in both groups. Amnesia during dental treatment is highly beneficial in reducing awareness at traumatic events for pediatric patients. Children who remember less about their perioperative events hold less psychologic trauma and are expected to present fewer negative behaviors.^28^ Although KET is characterized by its amnesic effect, this study showed no statistically significant differences between groups. However, we highlight that the number of children who exhibited anterograde amnesia was higher in the DEX-KET combination than in the DEX alone. This is consistent with a study by Singh, et al.^38^ (2014), in which a significantly higher number of patients sedated with KET exhibited anterograde amnesia when compared to patients who received DEX. It could be postulated that KET would only be able to induce profound amnesia when given in full dose. This was not the case in this study, in which the dose was reduced to half of that recommended, aiming to safely combine it with DEX. Moreover, we found that both groups had a similar profile for the incidence of drug-related adverse effects, which were all minor and easily treatable. Similarly, previous studies have reported minor procedural side effects in children sedated with DEX-KET combination or DEX alone.^12,35,36^ Control of stress in children during dental treatment is essential to ensure the delivery of proper dental care. It has been stated that s-IgA could be a potential stress biomarker for pediatric populations.^10^In this study, although mean s-IgA levels decreased following dental treatment as compared to pretreatment values, a significant difference has not been recorded. Likewise, mean differences between initial and final values of s-IgA were not found to be statistically significant between both groups. In this context, it could be assumed that children of both groups did not experience an event stressful enough to initiate an s-IgA response. In addition, any immediate stress that might have risen due to the procedure seemed to fade away in quite a short time in response to the drug administered, which in turn helped s-IgA levels to return to almost initial values. In accordance to this finding, two studies measuring s-IgA reactivity under acute stress found no s-IgA response to psychological stress before puberty. They suggested that children’s immune system may not respond to acute stress, in comparison to adolescents since children are born with an undeveloped immune system, which matures as they grow.^39,40^ The results of this study showed that neither drug regimen exhibited any effect on postoperative behavioral responses of children. This could be related to the capacity of the drugs to reduce pain and anxiety and reduce the awareness to the details of the treatment, all of which may influence the reduction of postoperative negative behavior. Dexmedetomidine shows neuroprotective effects and is capable of modulating the stress response, which may increase long term benefits.^41^ Moreover, ketamine possesses hypnotic, analgesic, and amnesic effects, which are important features that might be very beneficial in clinical practice.^42^ Another interesting aspect of ketamine is that it is thought to produce a unique clinical state by inducing a dissociation from the environment. This induces the patient into the classic “Ketamine stare,” in which the patient looks vacantly into space with open eyes and nystagmus.^42^ However, this phenomenon was not observed in this study. This might very well be attributed to the capacity of dexmedetomidine to reduce ketamine-induced dissociative symptoms, and produce sedation that rather resembles natural sleep.^4^ Similarly, a study by Sullivan, et al.^43^ (2001), who compared 2 oral ketamine-diazepam regimens in preschool children, reported that patients did not have the eye watering and pronounced nystagmus.

A possible limitation of this study was that s-IgA levels were measured at baseline and after completion of treatment but not during the procedure. A second limitation was that ketamine was administered without rendering it more tasteful by adding a palatable solution to increase drug acceptance. Further studies with more palatable preparations of ketamine and different concentrations should be studied to promote the use of buccally administered DEX-KET combination. Although all treatment procedures were accomplished in a profoundly anesthetized patient, standardization of dental treatment may be required in future studies to avoid any bias that may affect the study outcomes. Based on the previous data and within the limitations of this study, the hypothesis that there is no difference between DEX-KET and DEX administered via the buccal route regarding patient’s drug acceptance, child behavior during the procedure, amnesia, post-operative effects and procedure-induced stress as measured by s-IgA was rejected.

## Conclusions

- Both dexmedetomidine-ketamine combination and dexmedetomidine alone are effective in providing hemodynamic stability.
- DEX-KET shows a poor taste which might limit its acceptance in buccal administration.
- DEX-KET combination showed superior behavioral improvement during sedation sessions.
- Amnesia was comparable in both regimens with no significant adverse events.
- Salivary s-IgA is not considered a potential stress biomarker in sedated children.
- Subjective post-operative questionnaire assessment revealed no negative effect on behavior 24 hours after the sedation session in both groups.
